# A New Roadway Eventual Obstacle Detection System Based on Computer Vision

**DOI:** 10.3390/s20185109

**Published:** 2020-09-08

**Authors:** Mariano Gonzalez-de-Soto, Rocio Mora, José Antonio Martín-Jiménez, Diego Gonzalez-Aguilera

**Affiliations:** Cartographic and Land Engineering Department, University of Salamanca, Hornos Caleros 50, 05003 Avila, Spain; marianogds@usal.es (M.G.-d.-S.); rociomora@usal.es (R.M.); joseabula@usal.es (J.A.M.-J.)

**Keywords:** obstacle detection, animal crossings detection, roadway, computer vision, software development

## Abstract

A new roadway eventual obstacle detection system based on computer vision is described and evaluated. This system uses low-cost hardware and open-source software to detect and classify moving elements in roads using infra-red and colour video images as input data. This solution represents an important advancement to prevent road accidents due to eventual obstacles which have considerably increased in the past decades, mainly with wildlife. The experimental evaluation of the system demonstrated that the proposed solution detects and classifies correctly different types of moving obstacles on roads, working robustly under different weather and illumination conditions.

## 1. Introduction

In the past decades there has been a large increase in road accidents due to animal crossings. Bruinderink and Hazebroek [1] estimated the annual number of wildlife-vehicle collision (WVC) in Europe (excluding Russia) at 507,000, which resulted in 300 people killed and 30,000 injured, and material damage amounting to $1 billion (U.S.). In Spain, according to the general direction of traffic statistics [2], there are about 14,000 animal-vehicle collisions (AVC) per year (66.3% with wild animals and 37.7 % with domestic animals) with a fatality rate around 3%. With respect to the WVC, wild boar and roe deer are the most problematic species in Spain (79% of reported accidents). These AVC represent the 8.9% of the total traffic accidents in Spain with an annual economic cost of 105 million of euros [3]. Furthermore, Mehdizadeh et al. [4] and Hu et al. [5] present a review of the applications in road traffic safety analysing the crash risk in roads, including AVC and collision mitigation sensing systems.

The accidents with animals are too frequent with the corresponding safety problems for the vehicle travellers and the resulting economic and environmental consequences. Van der Ree et al. [6] found numerous papers that expose the problems associated with AVC and analyse different animal mitigation methods based on reducing the presence of animals in the roadways and/or roadside. Rytwinski and Fahrig [7] analysed the problems caused by the road over the animal populations describing the current and future problems than these roads are generating, predicting possible future consequences and demonstrating that it is very important to install and use systems to prevent this type of road accidents. More recently, Wilkins et al. [8] analysed the animals-vehicle collision in Texas (USA), highlighting that most of the collisions are in the first and last hours of the day, for both, domestic and wild animals, demonstrating that it is very important to use efficient roadway animal detection systems (RADS) able to work under dark conditions. They also estimated the high economic costs of these collisions and they obtained that most of the crashes are with car vehicles, whereas the worse consequences are for motorcycles.

Van der Ree et al. [6] presented works that analyse animal mitigation systems, specifically the fencing method is very efficient to prevent WVCs. However, fencing all roadways with animals is too expensive and it would entail important consequences in the habitat and behaviour of the wildlife animals. Smith et al. [9] evaluated the use of crossing structures to allow the wildlife connectivity across roads combined with fencing, but it is a very expensive method which could be suitable only for specific cases, as migratory routes or similar. Furthermore, Glista et al. [10] reviewed other mitigation techniques, as ultrasound, olfactory repellents, road lighting, population control, habitat modification, etc., but all of them present problems: road lighting, population control, habitat modification methods have a very negative impact over animals, the ultrasound method only works with some species, the olfactory repellents have a very limited durability.

On the other hand, there are other approaches based on RADS, which detect the animal presence in the roadway and alert the driver. This type of system does not add additional impact over the animal habitat neither the road. Numerous works can be found that analyse the use of RADS. Huijser et al. [11] and Huijser et al. [12] described, analysed and compared different RADS installed in USA and Europe. Huijser et al. [13], Grace et al. [14], Grace et al. [15] and Gagnon et al. [16], studied the effect of RADS over the driver’s behaviour, concluding that the effect of installing RADS is very positive. However, all authors agree that the accuracy of RADS should be improved. These works describe and analyse different methods: from a series of infrared sensors placed at some meters intervals on both sides of the road to detect the body heat of large animals or microwave radar sensors able to detect large animal movements from some meters. However, the main limitations about both systems is the presence of a lot of false positives. In addition, these systems are not able to know if the animals enter or exit.

In the last year, computer vision (CV) has experimented very important improvements. Some of this CV systems are based on thermal or infrared cameras. For instance, Zhou et al. [17] presented an application developed in MATLAB R2015a^®^ [18] to detect deer in mid-infrared (MIR) images. Forslund and Bjärkefur, [19] analysed a RADS based on far-infrared (FIR) to be boarded in cars, which provides a range until 200 m. However, MIR or FIR sensors are very expensive and greatly increase the cost of the system. Therefore, RADS based on CV are the most suitable option now, being possible to find very good and low-cost red-green-blue (RGB) and near-infrared (NIR) cameras and controllers. Price Tack et al. [20] present a MATLAB R2012b^®^ algorithm to detect animals, which works for species that are relatively monotone in ‘.jpg’ images. They obtained a success rate of 90%, but they required a manual processing time to improve the success rate and adjust some input variables in the algorithm. For its part, Mammeri et al. [21] and Jaskó et al. [22] described and analysed algorithms based on OpenCV [23] to detect animals on the road from RGB images. Their algorithms incorporate a subroutine to discriminate possible false positives training with images that can lead to error with the disadvantage of requiring a training and a dataset for each animal species. Dhulekar et al. [24] also present an algorithm to detect animals in RGB images using a training dataset. Their algorithm can be adapted to run in a Single Board PC, like Raspberry Pi 3 using OpenCV, but their results are presented in MATLAB 2015a^®^ using a PC. Trnovszký et al. [25] and Benraya and Benblidia [26] compared four methods to detect movement from video sequences, all of them are based on Gaussian mixture models (GMM).

Furthermore, other important problem to be considered is the collisions between vehicles and any type of eventual obstacle. Sewalkar and Seitz, [27] present a review of the systems to prevent this type of collisions with special emphasis on vehicle-pedestrian collisions. Other authors have developed solutions based on machine learning (ML) or deep learning (DL): Algabri and Choi, [28] present a method to detect and track people in indoor environments, whereas Qiu et al. [29] focused on different types of moving obstacles in outdoor environments. More recently, other authors, Chang et al. [30], Yang et al. [31] and Qiu et al. [32] have developed solutions for obstacles detection for self-driving cars. However, the main drawback of these solutions based on ML/DL is that, most of them, require a lot of training and thus existing data.

Considering this growing literature in the field of RADS and eventual obstacles vehicle collisions, this paper presents the development of a new static roadway eventual obstacle detection system that can be used like a RADS with additional features to discriminate between animals, pedestrians or cyclists, estimating the moving obstacle speed. Therefore, this system can also be installed in problematic urban roads to prevent collisions with pedestrians or cyclists. The system encloses two main parts: (i) a low-cost RGB and NIR camera and (ii) a software developed based on computer vision algorithms. This system guarantees, in comparison with those developed previously, the following advantages: (a) It combines different CV strategies to solve the weaknesses previously remarked, especially in reliability; (b) it does not need training; (c) it is independent of the obstacle type and different type of obstacles can be detected; (d) it can be configured quickly to solve different scenarios (e.g., interurban roads or even urban roads); (e) it works both during day and night; (f) it is based on a low-cost hardware and an open-source software. 

This paper has been structured as follows, after this introduction where a brief state-of-the-art has been outlined, Section 2 describes in detail the materials (hardware) and the method (software) developed. Section 3 outlines the experimental results obtained in outdoor urban scenes under different illumination conditions. A final conclusion section is devoted to highlight the impact of the system and the future improvements. 

## 2. Materials and Methods

### 2.1. Hardware Architecture

**Figure 1:**
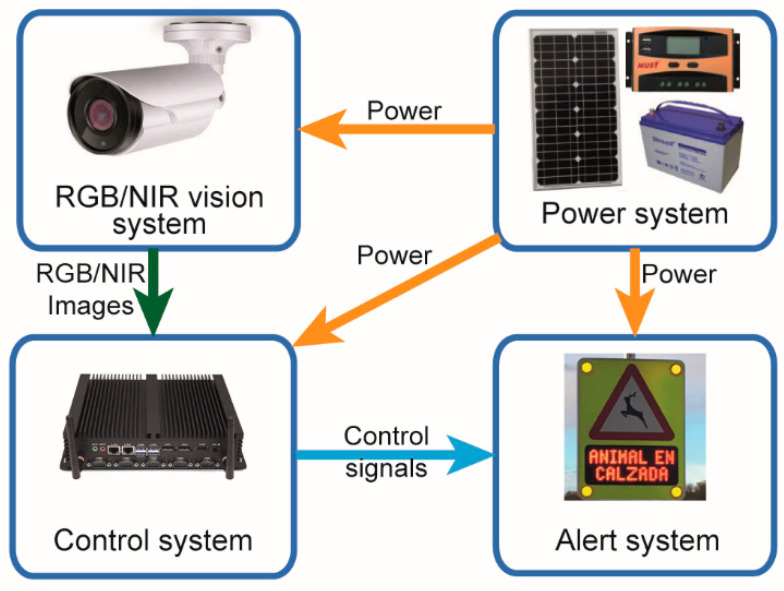
Hardware architecture.

The hardware architecture designed is outlined in Figure 1. It is formed by an optical vision system to capture the images, an alert system to warm the drivers, a control system to process the images and manage the alerts and a power system to provide energy to all of them. These systems are briefly described below. 

#### 2.1.1. Vision System

Most of the AVC happen in low light hours, as can be found in the results of the works exposed by Wilkins et al. [8]. Therefore, it is very important to use a vision system able to work under dark conditions. Furthermore, Pinchon et al. [33] evaluated the relevance of four different spectral bands under adverse weather conditions: spectral band 0.4 to 0.65 μm (visible RGB), 0.4 to 1 μm, 0.6 to 1.7 μm and 8 to 12 μm and they obtained that the longest wavelength is the most robust against adverse weather conditions and that the NIR systems are better than visible RGB vision systems. Since FAR systems (long-wave infrared) are too expensive, our system is based on a NIR vision system.

The system uses a RGB/NIR camera (see Table 1), model FS ‘FS-VCBOZ-4M’ [34]. This camera can provide a maximum resolution of 2560 × 1440 pixels up to 20 FPS and a minimum resolution of 352 × 288 pixels (in triple image mode) up to 25 FPS in H.264, H.265 and MJPEG formats. It incorporates a Hi3516D processor with an autofocus system and automatic adaptation between RGB and NIR vision mode. The price of this vision system is around 100 euros [35]. We can find many cameras with these features and a similar price, but a camera with an image sensor specifically designed for CV could cost several hundred or, even, thousands of euros [36].

#### 2.1.2. Control System

The control system must be able to activate the alert system with high resolution (near to HD resolution) in RT with a speed of 6 FPS, which is enough for the correct work of the algorithms (e.g., background subtractions, tracking, etc.,). Furthermore, this system must be rugged to work under adverse weather conditions (protected by a box).

The used system specifications are defined in the second column of Table 2, it is equipped with a powerful central processing unit (CPU) Intel Core I7 8550U, a fast RAM 4G DDR4 and a SSD of 128 Gb to store the program and the results [37,38].

Table 2 also outlines the features of two alternative control systems where we can see that the price is higher than the proposed system and that the main differences are in its ruggedization against blows and weather conditions [39,40,41,42].

#### 2.1.3. Power System

The power system consists of an energy system, based on off-grid photovoltaic panels. The size of this system should be designed for the specific location of the system, considering the maximum hours of sun and shades, as well as the size criteria for the accumulation system. The size of this accumulation system can be designed according to the worst day but thinking that the operation of this system is not critical, and it can be off some hours (Table 3). 

#### 2.1.4. Alert System

The main features of this system are shown in Table 4. This alert system consists of a traffic signal accompanied by four luminous signs and a text panel indicating the presence of eventual obstacles. Concretely, it is a P-24 warning signal accompanied by a panel text with the text ‘OBSTACLE ON THE ROAD’ and four LED spotlights that we can see in Figure 1. This system incorporates a luminosity sensor and a dimmer control to adapt the light intensity of these spotlights to the ambient luminosity.

### 2.2. Software

The software is based on OpenCV programmed in C++. This software, by default, includes standard values for its setup, but they can be also manually changed in a config file and then charged from this config file. It offers the possibility of saving the obtained results (data and images) to do a postprocessing analysis, allowing us to obtain statistics results. Note that these control parameters have predefined standard values which can be valid for most locations. However, the config file allows us to solve some particular situations: (**a**) Changes in the camera position or orientation; (**b**) improve the SW processing and HW usage; (**c**) solve problems of communication with the camera; (**d**) adjust times, filters and speed limits to different eventual obstacles. Anyway, since the position and orientation of the camera is fixed, these control parameters only have to be adjusted during the installation of the system, whereas the internal parameters of the camera (e.g., focus, lighting, night vision, etc.,) are self-adjusted. 

**Figure 2:**
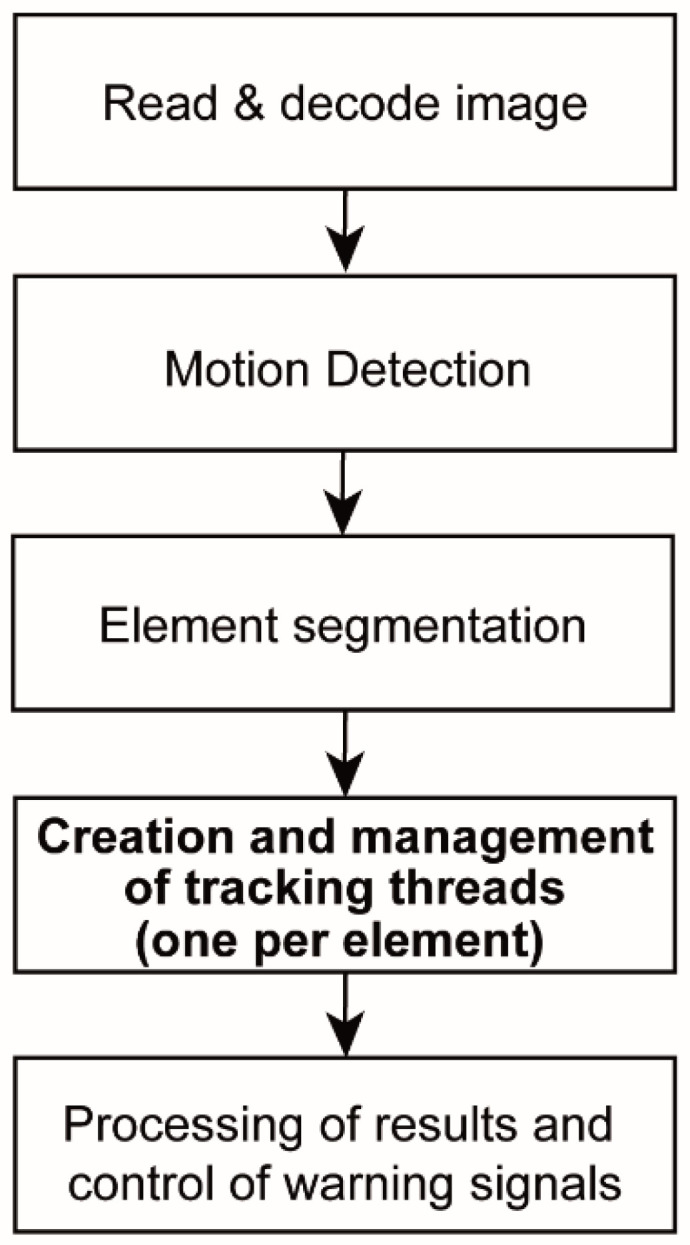
Software flowchart in the main loop.

The software developed has a main loop (Figure 2) to read images from the camera, detect moving elements, control the alert system and to manage children threads to track each detection. To track each one of the moving elements, a child thread is assigned [23,43].

The main loop, whose flowchart is showed in Figure 2, has the following steps:

1. Read and decode each image received from the camera and establish the region of interest (ROI) according to the parameters fixed in the config file.
2. Detection of motion based on background subtraction techniques. This process is described in Section 2.2.1.
3. Separation of the different moving elements within the ROI. This process is also described in Section 2.2.1.
4. Assign and manage a child thread to track each detected moving element, avoiding that the same element is followed and duplicated. In this part, the algorithm starts a child thread for each tracking loop and cancels it when the tracking element is lost. In Section 2.2.2 is described the tracking process carried out for each child thread.
5. Process the results returned by the children threads and control the states of the warning system for the drivers. The warning system is switch on when some elements are classified as obstacle, but when the obstacle disappears, the warning control remains activated for an ‘extra warning time’ (fixed in the config file) to prevent possible unwanted losses of tracking elements (e.g., due to large twist of the animal). In any case, we prioritise the presence of false positives over false negatives.

#### 2.2.1. Elements Detection

This process encompasses the second and third blocks of Figure 2 which are subdivided into two steps, detection and filtering, to obtain the next four steps:

1-a Background subtraction: this step creates a binary image which defines if each pixel belongs to the background or not. For it, we have considered and tested different background subtraction methods such as: (a) Mixture-of-gaussian (MOG) [44,45] which is based on gaussians mixture probability density functions; (b) MOG2 [46] which is also based on gaussians mixture models but adapting the number of components for each pixel; (c) k-nearest neighbours (KNN) [47] which presents recursive equations that are used to constantly update the parameters of a gaussian mixture model and to simultaneously select the appropriate number of components for each pixel according to the nearest neighbour; and (d) Godbehere-Matsukawa-Goldberg (GMG) [48] which combines a static background model (obtained with the first 120 images) with a probability estimation to detect foreground objects according to Bayes theorem. Although any of these methods can be selected in the config file, we worked with the MOG2 method because it produces very good results and it is very fast and adequate for real time applications. A comparative analysis of these methods can be found in [25].
1-b Filtering and corrections in the background subtraction: this step has two parts. The first one consists in joining the wrong segmentations in the moving element detections, by means of a dilation operator; and the second part eliminates those small size detections based on an erosion operator. Particularly, this erosion operator eliminates movements of background elements such as leaves, specks of dust, snow or water drops, among others. The amplitude in each transformation of this step is defined in the config file.
2-a Segmentation: this step separates the different detections and returns the contour of each one. It makes possible to track each element separately using parallel child loops running by different threads.
2-b Segmentation filtering: in this step a routine calculates the perimeter of each detection in order to filter wrong detections. It eliminates those detections whose perimeter is out of a range (defined in the config file). For instance, some wrong detections such as trees, branches, or even small elements such as leaves, rain, snow, etc., that have ended up forming large and irregular shapes.

#### 2.2.2. Motion Analysis: Element Tracking and Classification

**Figure 3:**
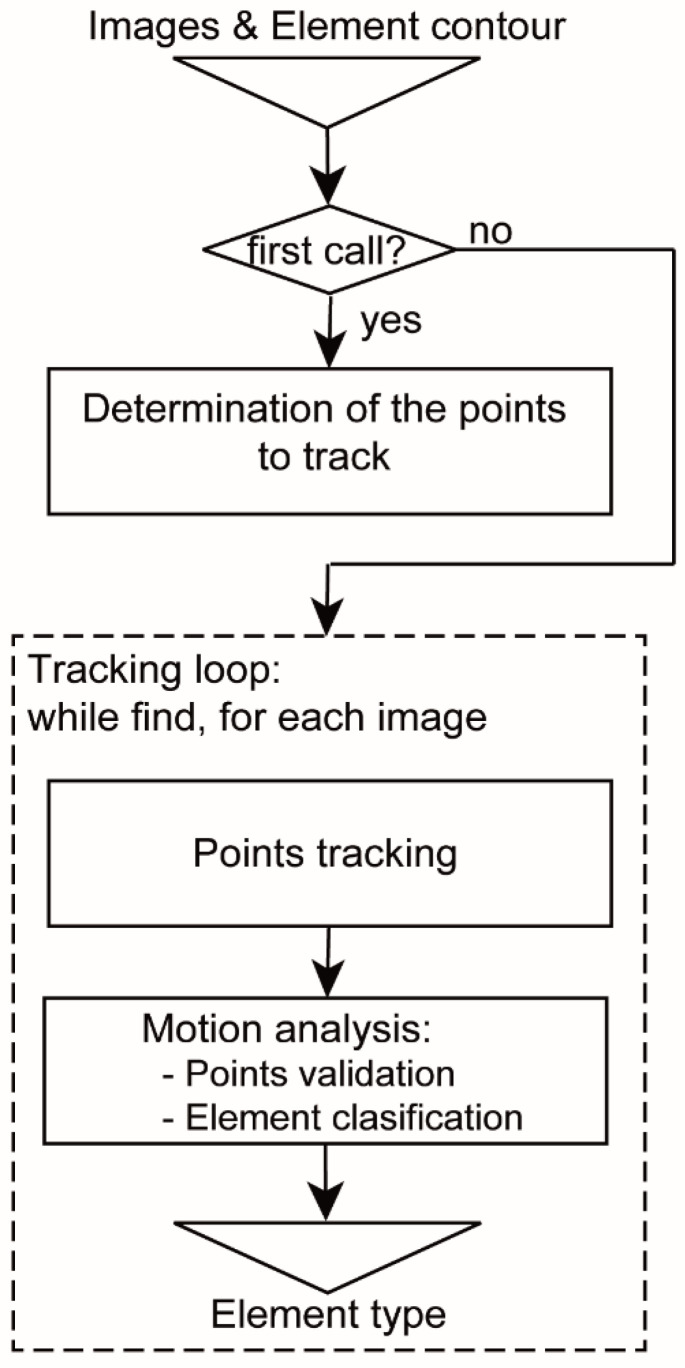
Software flowchart of each child thread.

The tracking and classification of each element is carried out by an independent subroutine executed by an independent thread. Each child thread receives the current and previous monochromatic image (grayscale or NIR) of the ROI and the current tracking element contour. Figure 3 shows a flowchart of each child thread. In the first step of each call, the child thread defines the feature points of the element using the ‘features from accelerated segment test’ (FAST) detector, [49]. If this detector does not find feature points because of different causes (e.g., blurry images caused by fog, rain, water or dirt on the lens), a more robust detector ‘good features to track’ (GFTT) is applied [50]. In Section 3.4.1 both methods are analysed and compared.

Next, a loop to track the feature points of the element and to analyse its displacement based on Lucas-Kanade (LK) algorithm [51] is executed, validating the type of the tracking element. In this step of motion analysis, the algorithm validates each new feature point and classifies the element type. This loop is running until a minimum number of feature points cannot be found and validated or until the element is discarded. In Section 3.2, the computational cost of these algorithms is analysed, whereas the efficiency of GFFT and FAST descriptors are studied in Section 3.4.1.

##### Validation of Feature Points

**Figure 4:**
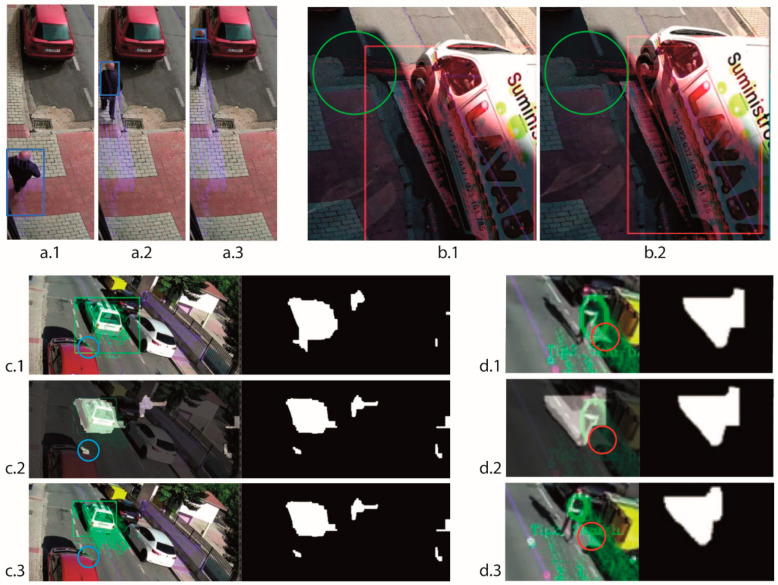
Validation of feature points: (**a**) Lucas-Kanade (LK) does not find matching, (**b**) the points displacement is very different due to a matching failure, (**c**) the points displacement is very different due to noise in the motion detection, (**d**) the points are out of the motion zone.

In this step, the algorithm analyses the displacement of each feature point between two consecutive images, discarding the point in the next cases:

- The LK algorithm returns a state value to indicate that the matching of the point in the new image is not good. For instance, in Figure 4a is outlined a sequence of images where the matching of LK is losing tracking points because of the variation in the perspective view of the element. We can see how the size of the bounding rectangle, in blue, is decreasing.
- The displacement between matching points is unusually different with respect to the typical displacement of the total feature points of the element. For instance, in Figure 4b is shown two consecutives images where the matching of LK of some tracking points, surrounded by a green circle, produces a very strange jump and then they are discarded. In Figure 4c we can observe how some tracking points are discarded because they are caused by a reflection and do not follow the moving element although they are in a movement zone that has detached itself from the moving element zone.
- The feature point is out of the motion zone defined by the motion detection loop, so it does not follow the element contour defined by the main loop (Section 2.2.1). For instance, in Figure 4d is outlined a sequence of images where some tracking points, surrounded by the red circle, are discarded because they are out of the motion zone (Figure 4(d.2)).

##### Element Classification

Each tracking element is classified according to the motion analysis. In this analysis the algorithm considers the element displacement in any direction and the element speed with respect to the axis of the road, and depending on the obtained results the tracking object is classified in the following types of elements:

- *Animals:* obstacles with an irregular path or stopped. They are represented as elements with a very low speed with respect to the axis of the road, but they move in any direction through the ROI. This trajectory has a displacement in some direction, not reciprocating movements typical of the effect of the wind over branches. They can be elements that has a fix position in the ROI, with small motions over its position, having been previously detected with a displacement path to enter in the ROI (e.g., animals stopped in the road or roadside feeding).
- *Pedestrian:* Moving obstacles with low speed in road direction, normally pedestrians. They could be also an animal, or a broken vehicle, which represent an obstacle in the road or even a broken branch or similar element dragged by the wind in the road direction.
- *Low speed vehicles*: cyclist, agricultural tractor, harvester, backhoe, etc. Elements whose travel speed measured respect to the axis of the road is high to be considered an animal or pedestrian, but it could increase the risk of collision accident. The speed range in this type of elements is also fixed in the config file.
- *Normal traffic*: cars, trunks, motorcycle, buses, etc. Elements whose travel speed measured respect to the axis of the road is very high. It is bigger than a value defined in the config file, normally 35 or 40 km/h.
- *Noise*: They are typically produced by the wind or by changes of illumination. These elements do not have displacement in the ROI, but they can have motion without changing its location. For example, the vegetation stirred by the wind, the own vibrations on the camera. These elements appear in the ROI detected by the main loop in the motion detection step, but they do not present a path to arrive to this location like the *‘animals.* Although, an animal cannot be detected by the tracking loop, it is unlikely that an animal does not generate a new path to be detected again by the system in the defined ‘extra warning time’ controlled by the main loop. When an element is classified as noise, it can be tracked again in the next iterations in order to reduce the false negatives.

To get the classification remarked above, the lineal speed of the tracking element is estimated, which allows us to analyse the driver’s behaviour when the warning signal is on and to get a statistical analysis of the traffic in that section of the road, between other possibilities. Numerous works can be found that presents different methods to estimate the vehicles speed with only one camera [52,53,54,55].

**Figure 5:**
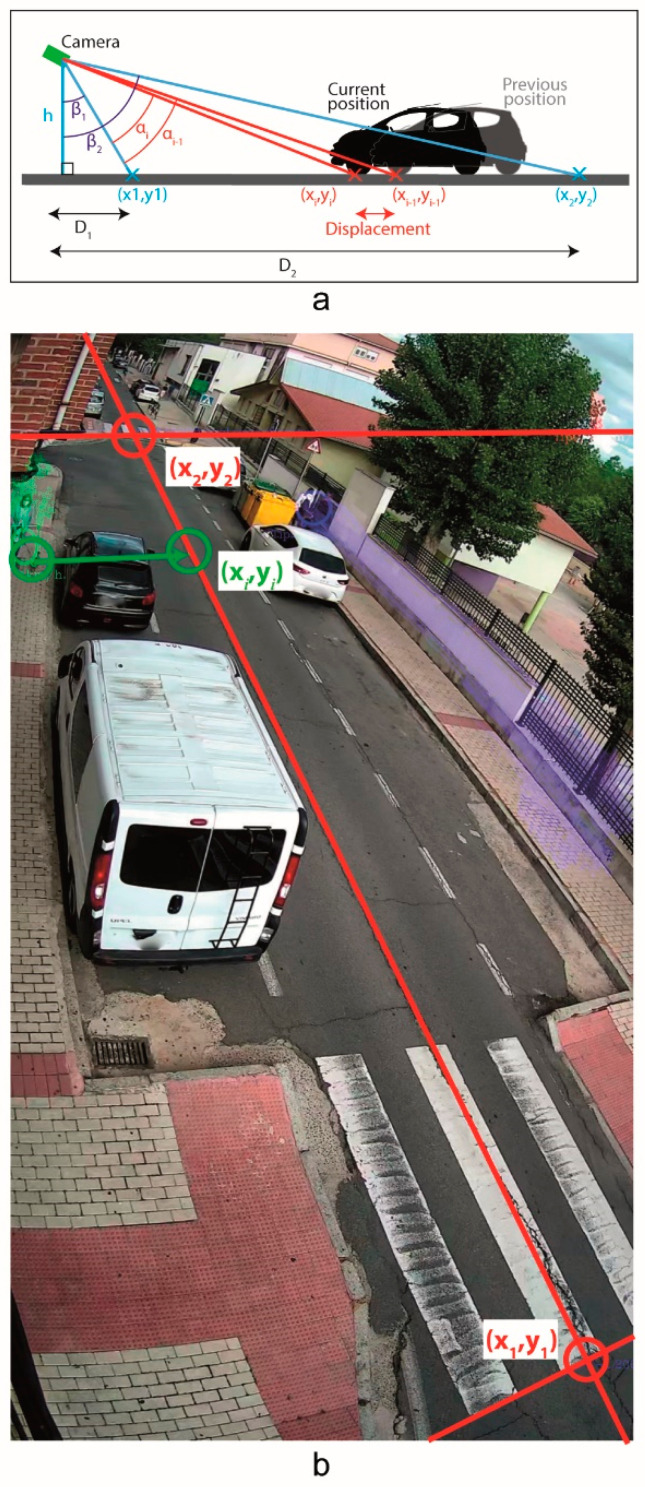
Linear speed estimation scheme. (**a**) graphic scheme of the profile view, (**b**) data over the test bench.

In particular, the methodology used to estimate the linear speed with respect to the road axis, projects the element position over the line corresponding to the road axis. Figure 5 shows the graphic representation of the parameters to estimate this linear speed. This process requires the following input data to calculate the speed:

- *h*: is the distance between the camera sensor and the road axis.
- (*x*_1_, *y*_1_): are the coordinates of the first pixel in the road axis within the ROI (Figure 5)
- (*x*_2_, *y*_2_): are the coordinates of the last pixel in the road axis within the ROI (Figure 5)
- *D*_1_: is the projected distance between the camera sensor and the point (*x*_1_, *y*_1_) along the road (Figure 5)
- *D*_2_: is the projected distance between the camera sensor and the point (*x*_2_, *y*_2_) along the road (Figure 5)

**Figure 6:**
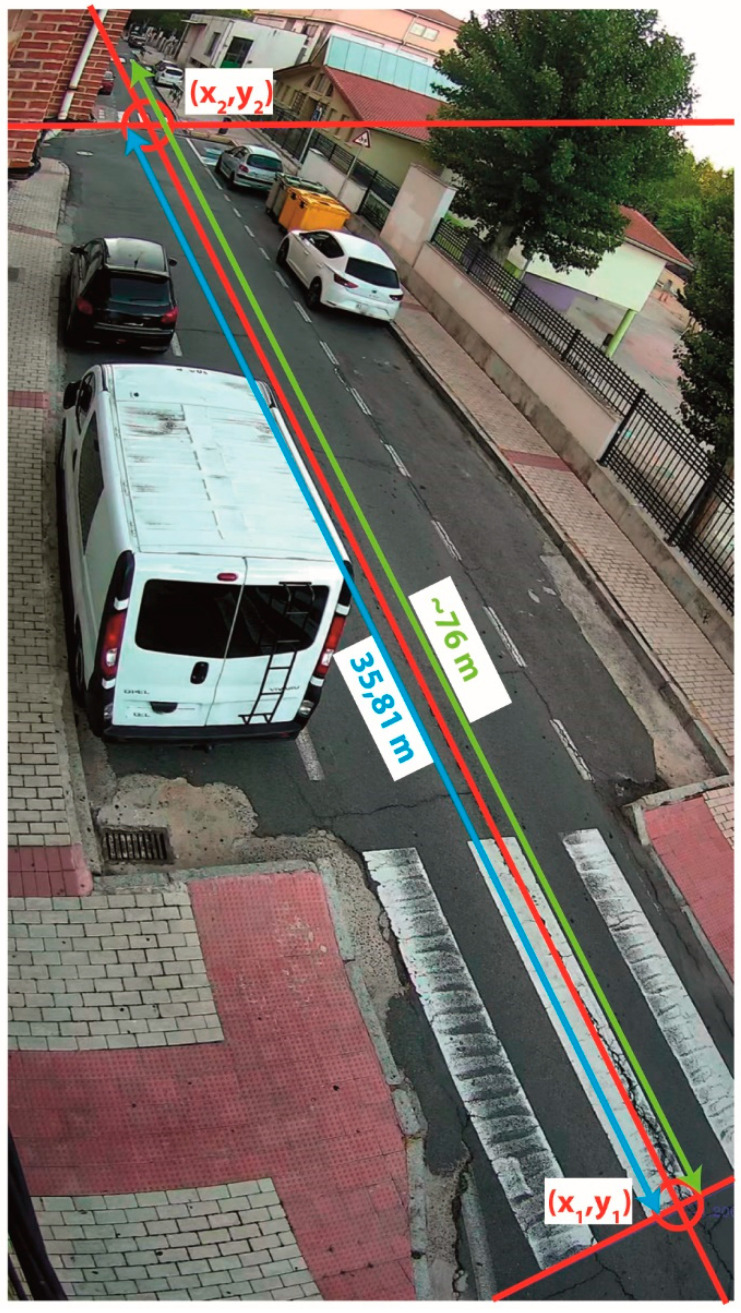
Experimental test bench.

These input data incorporate standard values once the camera position and orientation is adjusted using the grid that appears in the red lines of Figure 5 or Figure 6. An adequate standard configuration can be used in most locations, but these values can be also modified and loaded from the config file, which only would be necessary during the mechanical installation of the system. With these parameters we can obtain *β*_1_ and *β*_2_ which are the angles between the vertical of the camera and the line from the camera sensor to the points (*x*_1_, *y*_1_) and (*x*_2_, *y*_2_), respectively, (Figure 5). 

Knowing the value of *β*_1_ and *β*_2_ we can calculate the angle increment of each pixel of the road axis (red line in Figure 5b) to calculate *α_i_* and *α*_*i*−1_, corresponding to the current and previous position of the tracking element. Finally, the linear speed is estimated based on the displacement between the two consecutive images of which we know the timestamp (Δ*t*):

(1) $$v=\frac{h\left(tan\left(\alpha_{1}\right)-tan\left(\alpha_{i-1}\right)\right)}{\Delta t}$$

To get the pixel coordinates (*x_i_*, *y_i_*) we select a feature point of the element closer to the ground and, then, it is projected over the axis of the road (see green lines in Figure 5b). The pixel coordinates (*x_i−_*_1_, *y_i−_*_1_) would be similar but with the matching point of the previous image. The angle of this projection is set to a value at the point (*x*_1_, *y*_1_) and to other value at the point (*x*_2_, *y*_2_).For other points, it is considered a lineal variation of this angle along to the image.

Finally, the linear speed value is estimated doing the average during the last 3 s, where this time can be also modified in the config file. Furthermore, to solve the uncertainty of the method and to avoid oscillations between different types of moving elements, the system incorporates a hysteresis parameter (also configurable in the config file) that allows us to manage and filter the different types of moving elements.

## 3. Results and Discussion

In this section, the working of the system is assessed, examining each function developed in the software and analysing the capabilities of the system to detect and classify motion elements, using an urban road as case study under different visibility and weather conditions.

### 3.1. Experimental Test Bench

The tests were conducted over an urban road which we can observe in Figure 6. This road has vegetation that generates noise with the wind, circulation of cars, pedestrians, pets and bikes. Therefore, it was a good test bench before its final validation in an interurban highway.

The camera was positioned at a height of 5.15 m above the pavement and it was oriented to get a ROI slightly higher than 60 m (i.e., limit of the infrared camera) (Figure 6). Furthermore, we measured and calculated the necessary parameters, specified in Section 2.2.2 to estimate the speed over the road in order to classify the detected obstacles. 

### 3.2. Processing Hardware Validation: Times and CPU Usage

The hardware was assessed based on the use of CPU resources by the program in order to validate the working in real time and the appropriate rate of FPS with HD resolution (1920 × 1080 pixels). It is important to keep in mind that the lower the FPS, the greater the changes between consecutive images and, consequently, the less effective the background subtraction and the tracking algorithms. In this section we present the time assessment for the main thread (Figure 2) and for the children threads (Figure 3).

#### 3.2.1. Main Loop

In this analysis we have measured the processing time of each block program specified in Figure 2 processed by the main thread. In this timing analysis we have limited the maximum number of tracking threads to three values: 1 (the minimum value, very limited), 3 (an adequate value, to avoid blocking due to possible noise) and 8 (number of CPU threads, maximum recommended value). We have configured the detector FAST as the first option to define the feature points. Table 5 shows the obtained results, where ‘watch time’ is the real time spent by the control system and ‘CPU time’ is the sum of the time spent by each processor core (therefore, if the processor has 8 cores this time could be 8 times the ‘watch time’). It is important to consider the CPU time because it is directly associated with the number of CPU clock periods used by the algorithm and, therefore, it can be extrapolated to estimate the times using other processing systems with different cores number and clock frequency. All the times values are the average value obtained in one working day. We can see that ‘CPU time’ is slightly less than ‘Watch time’ because some parts of the process were paralleled by different cores. 

It can be observed that most of the values of time are very similar regardless the number of children tracking threads, except the value ‘Tracking thread management’ time which increases with the children number of tracking threads. This is because, in this step, the main thread must check each child thread to avoid duplicate tracking of the same element. The ‘CPU time’ is also near to the ‘Watch time’, in this step because ‘CPU time’ only considers the processor cores used by this thread, disregarding the ‘CPU time’ of the children threads. Other important factor to be considered is that to obtain the ‘Tracking thread management’ time, only the loops iterations were considered when there were child threads, because when there are no children threads present that means this time is disposable, much less than 1 ms. The times spent by the other steps of the main loop are independent of the number of tracking threads, because this number does not influence this step of the program and the small differences are due to the fact that each test is carried out at different times and, therefore, there are small changes in the treated images. 

The main data obtained from Table 5 is the maximum images rate of the system, which is calculated using the value of ‘Tracking thread management’ time when there are any tracking thread, to ensure the enough processing capacity in the worst case (when any tracking threads are working all the time). The obtained results show that this rate of images is more than enough to ensure an answer in time. The program needs less than 100 ms to process each image and for the tracking method, which is analysed in Section 3.4.1.

#### 3.2.2. Children Threads

We have carried out a similar times analysis for the children threads measuring the processing time of each block program specified in Figure 3 (Table 6). In this analysis we have also configured the detector FAST as the first option to define the feature points and we have limited the number of tracking threads to one. From Table 6 one can notice that the highest time is for determining the feature points which is, even, higher than the ‘Loop iteration’ time. This is because the step to extract the feature points only is executed one time for each detected element (in the first call of the tracking thread) and the ‘Loop iteration’ time is the average value of all thread iterations. The other important time is the ‘Points tracking’ time which entails the images processing time spent by LK algorithm. Furthermore, it can be observed a low difference between ‘Watch’ and ‘CPU’ time, due to the internal parallelisation process carried out by the system compiler. 

### 3.3. Motion Detection: Background Subtraction Method

**Figure 7:**
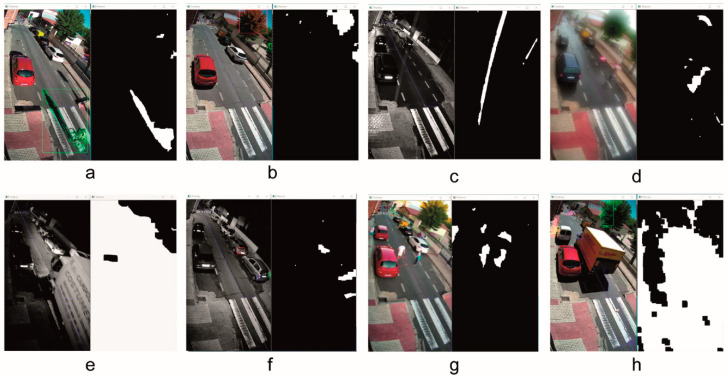
Background subtraction method: (**a**) sunny weather, (**b**) windy weather, (**c**) rainy weather, (**d**) light rainy fog, (**e**) very abrupt lighting change, (**f**) slight lighting change, (**g**) blur capture, (**h**) camera vibrations.

The motion detection was applied using the background subtraction method. Figure 7 shows some results obtained using MOG2 algorithm under different weather conditions. Note that the binary images represent the motion detection; white zones correspond to the motion elements and black zones to the background. The RGB and NIR images correspond to the camera capture and include the tracking data.

This method allows to avoid the shadows detection, however in our test we prefer to detect shadows to reduce the probability of false negatives. For instance, in Figure 7a we can see the result obtained in a sunny day, in the last hours of the day, where a big shadow of a cyclist moving over the road can be seen.

With respect to the noise generated by the wind, Figure 7b shows two detections in the movement of the branches of some trees. In this case the blurred applied over the image is not enough to compensate the vibrations of these branches, but they are discarded in the motion analysis process because it is an element with an oscillating movement, without path of entry in the ROI. We have a similar problem in Figure 7h where a large vehicle generates big vibrations in the camera, which are not compensated by blurring. But in this case the motion zone is so big that the element is filtered in the segmentation process and it does not pass to the motion analysis. As for the blur setting, Figure 7g shows the result of motion detection in an unfocused capture. Here we can see that this algorithm works very well with blurred images and the results are good. Even under adverse vision conditions such as dense fog (Figure 7d), the system provides a correct motion detection.

Figure 7c also presents results with adverse weather conditions. In this case it can be observed that some raindrops are detected by the MOG2 algorithm. These results appear mainly under low light conditions when the camera needs more time to get the frame and the raindrops are captured like little grooves of water, as can be seen in Figure 7c. However, this is not a problem for our system because they are quickly filtered.

Finally, in this motion detection we have analysed the problems caused by lighting changes. Figure 7e shows the result of a very abrupt lighting change caused by the lights of a vehicle in the middle of the night, but this motion detection is quickly discarded by the segmentation filtering. Furthermore, Figure 7f presents the result when we have slight lighting changes. Although these motion detections get to pass the program filters, they are later discarded in the motion analysis because their displacement is null.

### 3.4. Motion Analysis

Once we have detected and segmented the motion elements, the next step is to analyse its movement to define the element type and to discard possible false detection. We have divided the test in two groups: (a) definition and tracking of feature points, (b) classification of the elements detected and tracked.

#### 3.4.1. Definition and Tracking of Feature Points

For the definition of the feature points we have considered and evaluated the two detectors: GFTT and FAST [49]. In the first tests we have analysed the processing times of the tracking threads using both detectors, showing the number of points detected by them. Table 1 outlines the average values of the obtained results during one working day. In this table can be noticed that GFTT takes almost five times more than FAST, providing fewer feature points. Furthermore, an interesting result is the time spent by the tracking method LK [51], which is similar for both detectors despite the big difference in feature points to track. This could reflect that the feature points provided by FAST are much easier to follow using LK method. With respect to the total iteration time, we obtained similar results because the weight of the definition is very low in the average value, being only executed one time by the motion element detection (Figure 3). Regarding the parallelisation of the internal instructions, it is very low for both detectors, because we can see in Table 7 that the values of the ‘Watch’ and ‘CPU’ times are similar.

**Figure 8:**
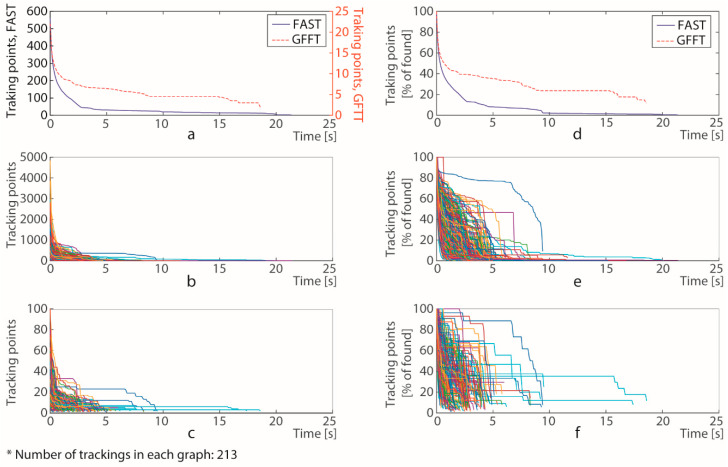
Time evolution of tracking points using GFTT and FAST detectors: (**a**) average number of tracking points using FAST and GFTT, (**b**) number of tracking points using FAST for each tracked element, (**c**) number of tracking points using GFTT for each tracked element, (**d**) average percentage, with respect to the initial value, of tracking points using FAST and GFTT, (**e**) percentage of tracking points using FAST for each tracked element, (**f**) percentage of tracking points using GFTT for each tracked element.

Other important factor to be considered is the effectivity of the matching for the tracking of the feature points defined with both methods. Figure 8a shows the evolution in the number of feature points found by LK method for both options to detect the initial feature points over the same element. 

**Figure 9:**
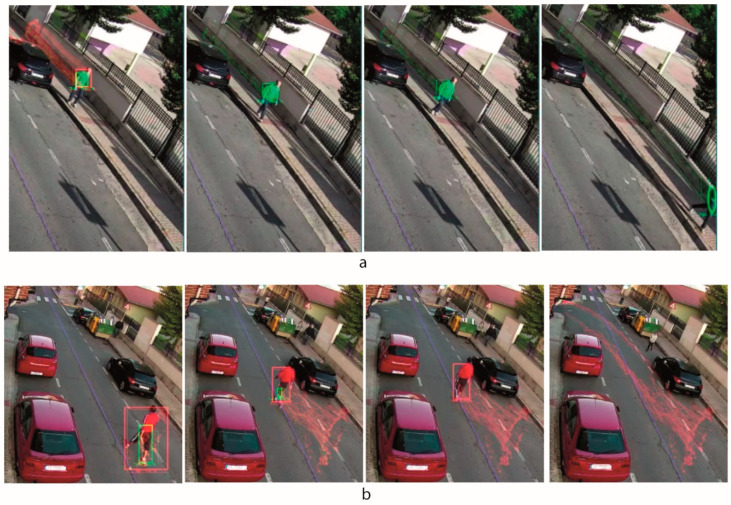
Singular cases of the tracking of features points defined with both detectors, GFTT and FAST: (**a**) sequence of the evolution in the feature points where the tracking using FAST detector ends before, (**b**) sequence of the evolution in the feature points where the tracking using GFTT detector ends before.

In these graphs we have evaluated the tracking of 213 moving elements which are within the ROI for, at least, 3 s, and considering that the element is discarded when the number of found features points is lower than 3. Figure 8a shows the average number of tracking points with respect to the time and Figure 8d represents similar data considering the percentage of tracking points regarding its values at the start of the tracking, which are the feature points obtained by the corresponding detector. Here, it can be noticed that, at the beginning of the tracking, the loss of feature points is more pronounced in the case of FAST detector, but later is quite similar achieving longer tracking times in the case of points detected with FAST. Furthermore, in Figure 8a can be observed the large difference in the feature points defined by both detectors. Figure 8b,c represents the individual number of tracking points with respect to the time for each of the 213 moving elements tracked by the program. Similarly, Figure 8e,f represents this equivalent data considering the percentage of points regarding the number of feature points obtained by the corresponding detector. In these four graphs (Figure 8b,c,e,f) it can be seen that the difference in the tracking time for each element is not so large as the number of feature points but it is bigger for the points defined with FAST. The average values of time are 4.63 and 2.47 s when we use FAST and GFTT, respectively. Furthermore, in the 84.0% of the cases analysed, the tracking using GFTT detector ends before than the tracking using FAST (Figure 9b), and only in 4.7% of the cases it is the other way around (Figure 9a).

The main conclusion from the graphics outlined in Figure 8 is that GFFT provides better points to track with LK, because the probability of losing points is lower; however FAST provides many more points and allows to decrease the probability of losing moving elements because it takes a longer time. 

**Figure 10:**
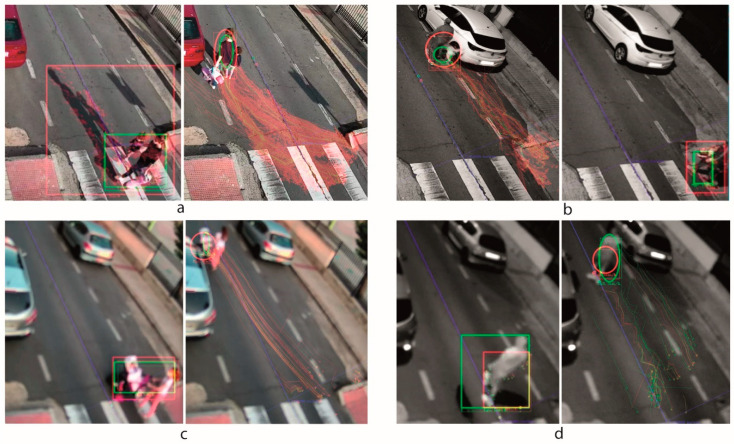
Tracking of points defined by FAST (red) and GFTT (green) detectors: (**a**) with good visibility during the day, (**b**) with good visibility at night, (**c**) adverse visibility conditions during the day, (**d**) adverse visibility conditions at night.

Other important aspect to be considered is the operation under different visibility conditions, caused by illumination and different weather conditions. Figure 10 shows some examples of feature points detection and tracking applying both detectors over the same moving element. Red lines correspond to the results using FAST and green lines to the results using GFTT. In Figure 10 we can see results applied for couples of frames; the left one is the initial feature points definition, in the moving element, and the right one is the tracking results after 3 s. In Figure 10a it can be observed that the results with sunny weather where we can see that FAST finds more points, even in the shadows. GFFT does not detect feature points in the shadows, resulting in a much smaller points area. Figure 10b shows the results at night, where FAST also provides a larger tracking area and both get to track and classify the element. Figure 10c,d also compare the results for day and night conditions, but when the visibility conditions are not good (e.g., raining, fog, etc.,). It can be noticed that the point distribution area is quite similar for both definition detectors and even larger in the case of GFTT as is shown in Figure 10d.

With respect to this analysis in different visibility conditions, Table 8 outlines the main factors that are affected by visibility conditions. Here we can see that FAST is much more vulnerable to poor visibility conditions, the average value of features points decrease to 95.3%, significantly reducing the computation time (‘CPU definition time’). Although the processing time (‘CPU tracking time’) for the matching by optical flow using LK is slightly increased in the case of less sharp images due to the worse visibility conditions. FAST gets to classify the moving obstacles more frequently in any case (95.8% of the analysed cases with good visibility and 91.0% with bad visibility, with respect to a 26.8% and 57.6% respectively using GFTT). Therefore, the program needs to re-detect and track the same element frequently when GFTT is used and, in any case the tracking time is longer using FAST than GFTT. Other interesting data is that whenever FAST finds features points, GFTT finds points to start the tracking, but not the other way around, especially with bad visibility where we have obtained that in a 40.4% of the total number of elements tracked GFTT detects enough feature points to start the tacking and FAST does not do it. Furthermore, there are a 10.2% of the detected obstacles where GFTT classifies the element as an obstacle and FAST does not detect enough features points to track the element. Therefore, with bad visibility conditions there are many cases where GFTT works better than FAST. In consequence, this system applies FAST as the first option, and if it does not find enough features points to start the tracking then GFTT is applied.

#### 3.4.2. Classification of the Elements

The classification process allows us to provide a semantic categorisation of the moving elements based on its path and speed. The speed is measured with respect to the axis of the road, in order to discern if it is a car, a low speed vehicle or a cyclist, a pedestrian, an animal, or some noise. Table 9 shows the results in the assessment of the speed calculation function. These tests were carried out using a bicycle equipped with an odometer that has an approximate precision of ±1 km/h. The second column outlines the average speed value read in this system along the stretch of road between the points (*x*_1_, *y*_1_) and (*x*_2_, *y*_2_) defined in Figure 6. The third column shows the speed values calculated analysing the video of the test manually, for which we have measured the time from when the bike passes through point (*x*_1_, *y*_1_) until it reaches point (*x*_2_, *y*_2_), both of them defined in Figure 6, as well as the distance between them. In this case we calculate the standard deviation for this speed value using an approximate precision time of ±0.2 s, which corresponds with the time measurement error of ±1 frame at 10 FPS at points (*x*_1_, *y*_1_) and (*x*_2_, *y*_2_). The fourth column reflects the speed values estimated by the algorithm developed during 3 s, that is the time defined in the config file to classify the tracking elements. Three seconds is enough to calculate a good average value (of 30 values in this case) without generating appreciable delays in the activation of the warning signal. In this case, the SD values, outlined in the fifth column, are calculated for each group of tests with the same reference speed. Finally, the sixth column reflects the percentage of error for each speed measurement regarding the average value of both references. Although, the speed values obtained are considered an estimation, they are valid enough to provide a correct performance of the system and thus to discern between eventual obstacles (very low speed) and traffic (high speed). 

**Figure 11:**
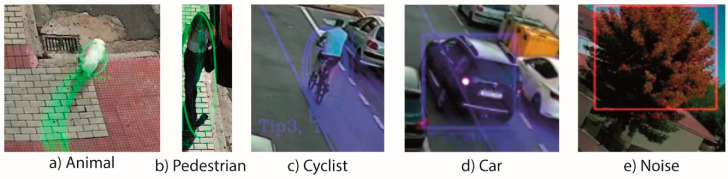
Classification of the elements: (**a**) animal, (**b**) pedestrian, (**c**) cyclist, (**d**) car, (**e**) noise.

Figure 11 shows an example of each classified element type, where lines correspond to the tracking of the features points, the bounding box to elements in evaluation or elements which are not obstacles (cars or noises) and the bounding ellipses to elements classified as obstacles (e.g., animal, pedestrians, or low speed vehicles). Considering that the speed values obtained are an estimation and values of the error column, whose maximum value is 16% with a standard deviation (SD) of 5.3%, the default value of the hysteresis parameter to change between elements types has been set at 30% of the speed limit, which is higher than the sum of the maximum value and the SD of this error column. Here we can observe that the system is able to detect animals, pedestrians and cyclists when they are not in the limits of the criteria. Therefore, it can be used not only like a RADS, but also to prevent collisions with other eventual obstacles in any type of road, including urban roads where the visibility of the drivers could be complicated. In any case, it is clear that the system achieves the main purpose with good reliability, which is to discern between eventual obstacles (very low speed) and traffic (high speed).

### 3.5. Analysis of False Negatives

**Figure 12:**
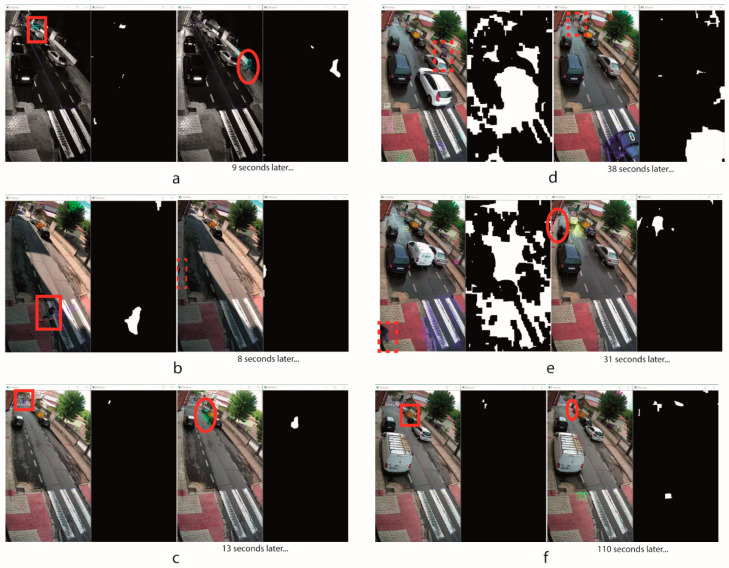
Cases of false negatives: (**a**) case 1, (**b**) case 2, (**c**) case 3, (**d**) case 4, (**e**) case 5, (**f**) case 6.

Considering the importance of avoiding false negatives versus false positives, since the former increase the risk of an accident, Table 10 outlines the most relevant cases of an analysis of false negatives detected during 16 working hours of the system, considering different tests with different weather and illuminations conditions (sunny, cloudy, rainy and at night) with a total number of 27 false negatives. In this period the system detected 175 positives of which 11 were false positives. Figure 12 shows an example of each case of false negative defined in Table 10. In this figure the red rectangles identify obstacles in the classification process, the dashed red rectangles identify non-detected obstacles and the red ellipses identify detected obstacles. To define each false negative, a visual analysis of the captured images was used as reference, considering false negatives only when the eventual obstacles are within the ROI more than 6 s, that is the double of the time fixed to classify each element. In Table 10 it can be observed that the most of false negatives take a short time (cases 1, 2 and 3), and that many of them are finally classified (cases 1, 3, 5 and 6). When they are not classified, normally they are elements which appear during a very short time within the ROI (case 2). In most cases the tracking process does not achieve to classify the element in the first attempt because it loses the feature points and need additional attempts (each number of ‘Time during which the element is tracked (s)’ column corresponds to one attempt) and, therefore, sometimes the element disappears in the ROI before being classified. These false negatives do not represent a large problem because the time is very short (<15 s). However, when the eventual obstacle appears together with a lot of noise, like it is shown in Figure 12d,e, it is not possible to define the element contour and the false negative is longer (cases 4 and 5).

The worst case is when the element is not classified while enters within the ROI, being later considered as noise because it does not have displacement within the ROI (case 6). Fortunately, the likelihood of these false negatives cases is very low. Only a 3.7% of the false negatives detected in this analysis, and its duration was not very long (110 s), because it is very unlikely that this type of element stays in the same position for a long time.

The total number of false negatives represent a 14% of the total positives, however, with respect to the time, the duration of the false negatives is lower than a 1% of the total time of the tests. Therefore, according to these results, the likelihood of a false negative is very low.

**Figure 13:**
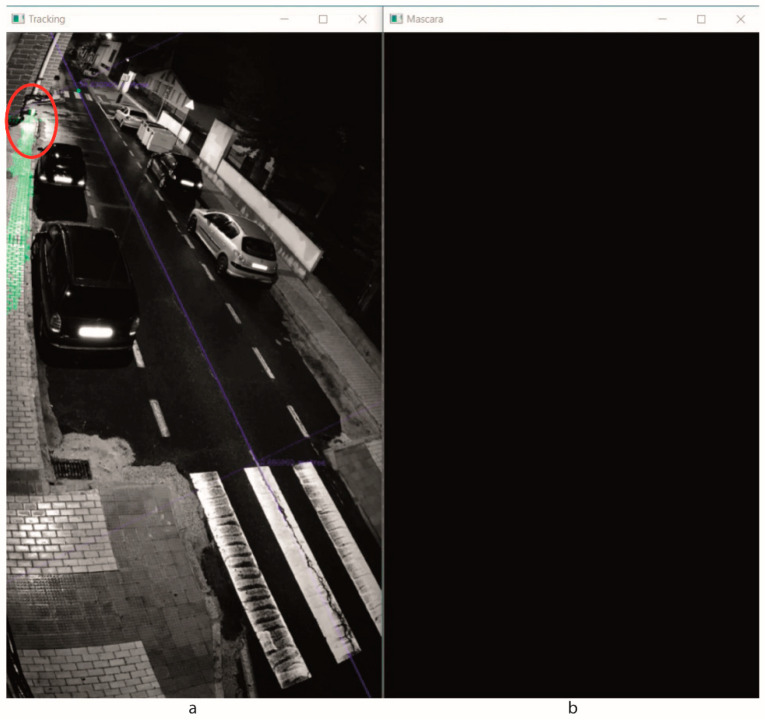
Typical case of false positive: (**a**): NIR image captured by the camera with the tracking data. (**b**): binary image of the background (black zone).

With respect to the false positives, most of them are caused by a wrong matching in the element tracked, which matches some points of the background with the tracked points when an eventual obstacle disappears of the ROI (see Figure 13). Nevertheless, this situation is corrected in a few seconds because these wrong feature points are out of the motion zone. 

## 4. Conclusions

This paper describes a new static roadway system designed to detect eventual obstacles and to prevent accidents. Particularly, an infra-red-based computer vision system that detects and classifies moving obstacles in order to trigger an alert in case some potential risk is proposed. The system is able to work with different illumination and weather conditions (sunny, cloudy, foggy, at night) using RGB and NIR images as input data. The NIR vision can work in a relatively high range of distances, up to 60 m in clear nights, improving the quality of the images when there is fog. The cost of the hardware is around 600 euros, which can be assumed as a ‘low-cost system’ by most of the companies that are dedicated to road maintenance.

In the experimental results we obtained that it is necessary a rate of 6 FPS for the correct tracking of the moving elements like pedestrians or animals, therefore we need to use a powerful CPU to process the HD images in RT without using a GPU. Particularly, an i7 processor of 8th generation for processing 12 FPS with HD resolution (1920 × 1080 pixels) using the proposed CV techniques would be desirable.

With respect to the motion detection or background subtraction, the techniques implemented offered very good results. We have obtained, by the experimental visual analysis, that most of the motions are detected, adjusting very well the contour of the moving element. In particular, the implemented method, MOG2, provides results that allow to detect the moving elements in the roadway under different weather and illumination conditions to reduce the probability of false negatives. In addition, this system tracks and analyses the motion of each element combining two detectors, GFTT and FAST, and applying the LK method to track these moving elements. GFFT provides points with lower loss probability in the tracking with LK and works better in bad visibility conditions. For its part, FAST provides many more points faster and with an easier tracking with LK, consuming less CPU resources, decreasing the provability of losing the moving elements and increasing the probability of obtaining an adequate point for the speed estimation. Although FAST does not work very well with bad visibility conditions, then GFFT is applied. The presented methodology to the motion analysis is based on determining the displacement of the element in any direction and the lineal speed in axis road direction to classify the motion element in: animals, pedestrians, cyclists or low speed vehicles, normal traffic or noise. The speed estimation method is based on the analysis of road displacements between consecutive frames, taking the road axis as reference and provides an estimation valid enough to classify the mentioned moving elements when they are not in the critical discrimination zone. The proposed algorithm carries out adequate filtering of tracking points to reduce the probability of false positives, typically caused by the wind or by illumination changes, and it is able to detect and classify any moving element under different weather conditions combining the background subtraction with the analysis of the displacement of the feature points to reduce probability of false negatives. Furthermore, we have reduced the likelihood of false negatives, especially when the eventual obstacle remains some minutes within the ROI, obtaining only false negatives when the eventual obstacle is within the ROI during short periods (<10 s) because the element is classified with each new movement.

Definitely, the presented system has been tested under different weather and illumination conditions, demonstrating detects and discerns between eventual obstacles (at very low speed) and traffic (at high speed), which is the main purpose of the system. It also classifies any eventual obstacle quickly and can be used in urban and inter-urban roads.

In future works, the system will be installed in an interurban road to thoroughly analyse its relevant aspects in a real environment during a longer time. In this future location, we will intensify the analysis described in this work to obtain more robust results in aspects such as: the internal computational load, the variable weather conditions, the classification accuracy, among others. Finally, we will conduct additional analysis to obtain the system performance based on the location of the obstacle with respect to the ROI and the camera position.

## Figures and Tables

**Table 1:** Camera features. Source: [35].

Feature	Value
Brand	FS
Model	FS-VCBOZ-4M
Image Sensor	OV4689
Effective Pixels	2688(H)*1520(V) (4Mpx)
Compression	H.264/H.265/JPEG/AVI/MJPEG
Video Delay	0.3S (Within the Lan)
Focus	Auto
Infrared Distance	60 m
Communication	TCP/IP, ICMP, HTTP, HTTPS, FTP, DHCP, DNS, DDNS, RTP, RTSP, RTCP, NTP, SMTP
Operation Temperature	−20 °C ~ +60 °C RH95% Max
Power Source	DC 12 V ± 10%, 1000mA

**Table 2:** Control system features and comparative with alternative commercial units. Source: [37,38,39,40,41,42].

Feature	Control System	Alternative 1: Control System	Alternative 2: Control System
Brand	WEIDIAN	BOXER	BELSATI
Model	WE63 8SJ-227XES	6640M-A1-1010	BS-B 500F
OS	Windows^®^ 10 Pro	Windows^®^ 10 IOT (64bit),	Windows^®^ 10 IOT (64bit),
CPU	Intel Core™ i7 8550U 4 Cores 8 Threads, 1.8 GHz up to 4.0 GHz	Intel^®^ Core™ i7-7700T, 4 Cores 8 Threads, 2.9GHz up to 3.8GHz	Intel^®^ Core™ i7-8700T, 6 Cores 12 Threads, 2.9 GHz up to 4.0 GHz
RAM	4 GB DDR4	4 GB DDR4	4 GB DDR4
Storage	128 GB SSD	128 GB SSD	256 GB SSD
GPU	Intel UHD Graphics 620	HD Intel^®^ 630	UHD Intel^®^ 630
Communication	Gb LAN 802.11ac Wi-Fi Universal Mobile Telecommunications System (UMTS)	Gb LAN Universal Mobile Telecommunications System (UMTS) GPIOs	Gb LAN GPIOs
Power	DC 12 V–19 V/6 A	DC 9–36 V	DC 9–24 V
Operating Temperature	−10~60 °C	−20 °C~50 °C (according to IEC68-2-14)	0 °C ~ 40 °C
Anti-Vibration	-	2 Grms/ 5~500 Hz	-
Certification	-	CE/FCC class A	IP40
Price	540 €	1239 €	1475 €

**Table 3:** Basic specifications of the power system.

Feature	Value
Power source	Photovoltaic panels
Energy storage	AGM or Gel Batteries
Autonomy	2 days
Power	12 V/170 wp

**Table 4:** Alert system features.

Feature	Value
Signal type	P-24
Warning	4 Dimmable LEDs & Text screen
Sensors	Photocell
Control	Digital input
Power	12 V, 20 W

**Table 5:** Main loop program times.

Maximum Number of Tracking Threads	1		3		8	
Times (ms)	Watch	CPU	Watch	CPU	Watch	CPU
Image reading and decoding	24.27	24.46	23.52	24.03	24.16	24.44
Motion detection and filtering	27.03	27.53	27.62	28.11	26.98	27.48
Elements segmentation and filtering	1.17	1.55	1.10	1.49	1.18	1.53
Tracking thread management	24.56	25.06	29.85	30.34	35.60	36.11
Results processing	0.16	0.17	0.16	0.16	0.27	0.28
Total program Cycle	77.20	78.76	82.26	84.14	88.20	89.83
Maximum images rate (FPS)	12.95	12.70	12.16	11.89	11.34	11.13

**Table 6:** Child tracking threads times.

Times (ms) *	Watch	CPU
Definition of feature points	15.90	16.42
Points tracking	11.49	12.00
Motion analysis	0.94	1.12
Loop iteration	12.43	13.11

* Feature points to track (average value): 351.98.

**Table 7:** Times analysis of the tracking threads using good features to track (GFTT) and features from accelerated segment test (FAST) detectors.

Method	GFTT *		FAST *	
Times (ms)	Watch	CPU	Watch	CPU
Definition of feature points	68.32	68.82	14.47	14.95
Points tracking (LK)	11.79	11.79	11.74	11.73
Results analysis	1.58	1.58	1.61	1.61
Loop iteration (tracking + analysis)	13.36	13.36	13.35	13.35

* Feature points to track (average value); GFTT: 22.07. FAST: 560.24.

**Table 8:** Comparation between GFTT and FAST detectors under different visibility conditions.

	Good Visibility		Bad Visibility	
Feature	GFTT	FAST	GFTT	FAST
Initial feature points	22.07	560.24	8.98	26.38
CPU definition time (ms)	68.82	14.95	66.16	3.60
CPU tracking time (ms)	11.79	11.73	12.94	12.45
Cases where the tracking ends earlier, with respect to the total number of obstacles *	4.7%	84.0%	22.8%	69.0%
Cases where the elements are classified as obstacles *, with respect to the total number of obstacles *.	26.8%	95.8%	57.6%	91.0%
Cases where the detector finds enough points to start the tracking, and the other detector does not, with respect to the total number of elements tracked (obstacles, cars and noises).	2.6%	0.0%	40.4%	0.0%
Cases where the elements are classified as obstacles * and the other detector does not find points, with respect to the total number of obstacles *.	0.5%	0.0%	10.2%	0.0%

* An obstacle is an element that is within the ROI more than 3 s and it is classified as animal, pedestrian or low speed vehicle, using any of the detectors.

**Table 9:** Measured speed values, in km/h.

Case	Odometer	Video	Algorithm	Algorithm SD	Error *
1	23 ± 1	22.6 ± 1.7	22.4	0.7	1.6%
2	23 ± 1	23.4 ± 1.8	23.3		0.9%
3	23 ± 1	22.6 ± 1.7	21.8		4.5%
4	23 ± 1	23.4 ± 1.8	23.7		1.9%
5	23 ± 1	23.9 ± 1.9	22.9		2.2%
6	18 ± 1	18.2 ± 1.1	16.4	0.9	9.1%
7	18 ± 1	18.2 ± 1.1	16.4		9.4%
8	18 ± 1	18.4 ± 1.1	18.2		1.1%
9	18 ± 1	18.4 ± 1.1	16.3		10.5%
10	18 ± 1	19.0 ± 1.2	18.0		2.9%
11	13 ± 1	12.6 ± 0.5	10.9	0.9	14.6%
12	13 ± 1	13.7 ± 0.6	11.2		16.0%
13	13 ± 1	13.7 ± 0.6	12.4		7.4%
14	13 ± 1	13.6 ± 0.6	13.0		2.1%
15	13 ± 1	14.5 ± 0.7	12.4		9.8%
16	6 ± 1	6.1 ± 0.1	5.8	0.5	4.6%
17	6 ± 1	6.3 ± 0.1	5.4		12.6%
18	6 ± 1	6.3 ± 0.1	6.3		3.3%
19	6 ± 1	6.4 ± 0.1	5.3		14.6%
20	6 ± 1	6.4 ± 0.1	5.3		14.8%

* Error regarding to the average value of both references.

**Table 10:** Analysis of false negatives.

Case	Average Duration of the False Negatives ± SD (s)	Average Time during for the Detection of the Motion Contour ± SD (s)	Average Time for the Elements Tracked (SD ≈ ±1) (s)	Reason Why an Obstacle is not Classified	% of Similar Cases *
1	9 ± 3	9 ± 3	2; 1.5; 2	The tracking loses the element a few times before classifying it.	33.3%
2	8 ± 2	8 ± 2	1; 1.5; 2; 2	The tracking loses the element a few times, and it disappears from the ROI.	25.9%
3	13 ± 3	13 ± 3	3; 3; 3	The element is classified as noise because it is so far before classifying it.	18.5%
4	38 ± 8	24 ± 6	1.5; 2; 1; 1.5; 1; 1; 0.5; 0.5	The element appears together with a lot of noise and then, the tracking loses the element a few times and it disappears from the ROI.	7.4%
5	31 ± 6	19 ± 4	1; 2; 1.5; 2	The element appears together with a lot of noise and then, the tracking loses the element a few times before classifying it.	11.1%
6	110 ± 20 **	56 ± 10 **	1; 2; 3; ...; 3	The tracking loses the element 2 or 3 times, and later it is classified as noise.	3.7%

* Regarding a total of 27 false negatives detected. ** These values are an estimation, because only there was one case.

## Abbreviations

AVC	animal-vehicle collision
CPU	central processing unit
CV	computer vision
DL	deep learning
FAST	features from accelerated segment test
FIR	far-infrared
FPS	frames per second
GFTT	good features to track
GMG	Godbehere-Matsukawa-Goldberg
GMM	Gaussian mixture models
HW	hardware
KNN	k-nearest neighbours
LK	Lucas-Kanade
MIR	mid-infrared
ML	machine learning
MOG	mixture-of-gaussian
NIR	near-infrared
RADS	roadway animal detection system
RGB	red-green-blue
ROI	region of interest
RT	real time
SD	standard deviation
SW	software
WVC	wildlife-vehicle collision
